# Simulator Pre-Screening of Underprepared Drivers Prior to Licensing On-Road Examination: Clustering of Virtual Driving Test Time Series Data

**DOI:** 10.2196/13995

**Published:** 2020-06-18

**Authors:** David Grethlein, Flaura Koplin Winston, Elizabeth Walshe, Sean Tanner, Venk Kandadai, Santiago Ontañón

**Affiliations:** 1 Diagnostic Driving, Inc Philadelphia, PA United States; 2 Computer Science Department Drexel University Philadelphia, PA United States; 3 Center for Injury Research and Prevention Children's Hospital of Philadelphia Philadelphia, PA United States; 4 Perelmen School of Medicine University of Pennsylvania Philadelphia, PA United States; 5 Annenberg Public Policy Center University of Pennsylvania Philadelphia, PA United States; 6 Geography Department Rutgers University New Brunswick, NJ United States

**Keywords:** simulated driving assessment, on-road exam, machine learning, adolescent, child, support vector machines, humans, accidents, traffic, cause of death, licensure, automobile driving, motor vehicle, motor vehicles

## Abstract

**Background:**

A large Midwestern state commissioned a virtual driving test (VDT) to assess driving skills preparedness before the on-road examination (ORE). Since July 2017, a pilot deployment of the VDT in state licensing centers (VDT pilot) has collected both VDT and ORE data from new license applicants with the aim of creating a scoring algorithm that could predict those who were underprepared.

**Objective:**

Leveraging data collected from the VDT pilot, this study aimed to develop and conduct an initial evaluation of a novel machine learning (ML)–based classifier using limited domain knowledge and minimal feature engineering to reliably predict applicant pass/fail on the ORE. Such methods, if proven useful, could be applicable to the classification of other time series data collected within medical and other settings.

**Methods:**

We analyzed an initial dataset that comprised 4308 drivers who completed both the VDT and the ORE, in which 1096 (25.4%) drivers went on to fail the ORE. We studied 2 different approaches to constructing feature sets to use as input to ML algorithms: the standard method of reducing the time series data to a set of manually defined variables that summarize driving behavior and a novel approach using time series clustering. We then fed these representations into different ML algorithms to compare their ability to predict a driver’s ORE outcome (pass/fail).

**Results:**

The new method using time series clustering performed similarly compared with the standard method in terms of overall accuracy for predicting pass or fail outcome (76.1% vs 76.2%) and area under the curve (0.656 vs 0.682). However, the time series clustering slightly outperformed the standard method in differentially predicting failure on the ORE. The novel clustering method yielded a risk ratio for failure of 3.07 (95% CI 2.75-3.43), whereas the standard variables method yielded a risk ratio for failure of 2.68 (95% CI 2.41-2.99). In addition, the time series clustering method with logistic regression produced the lowest ratio of false alarms (those who were predicted to fail but went on to pass the ORE; 27.2%).

**Conclusions:**

Our results provide initial evidence that the clustering method is useful for feature construction in classification tasks involving time series data when resources are limited to create multiple, domain-relevant variables.

## Introduction

### Background

According to the Centers for Disease Control and Prevention, motor vehicle crashes (MVC) are the leading cause of death in adolescents aged 12 to 19 years in the United States [1]. MVC risk is disproportionately high among novice drivers, particularly those aged 16 to 17 years, and peaks immediately after licensure during the first months of unsupervised driving [2,3]. Previous studies have demonstrated that the majority of these crashes early in licensure are attributed to critical driving errors because of skill deficits, inexperience, and inattention/distraction rather than recklessness and deliberate risk taking [4-7]. Research has demonstrated initial evidence for the ability of web-based screening tests (eg, web-based assessment of cognitive impairments) to predict poor driving simulator performance [8]. However, there remains a critical need for a screening test to quantify skill level at the time of licensure to identify those who are underprepared to drive safely.

In 2017, a large Midwestern state’s driver licensing agency aimed to address the need to identify underprepared license applicants (likely to fail the on-road examination [ORE]) by developing and deploying a new portable virtual driving test (VDT) as a potential prescreen tool for ORE [9]. The VDT was built on the Ready-Assess platform [10] (Diagnostic Driving, Inc) and was designed as a safe, reliable, and portable method for evaluating novice drivers’ preparedness to respond to common and serious potential crash scenarios [11-13]. Ready-Assess incorporates and expands on the scenarios and metrics of the previously validated simulated driving assessment (SDA) [14] to provide a variety of traffic situations to manage that go beyond what is feasible to assess during an ORE.

### Objectives

The VDT assessment (which lasts approximately 7 min) includes both high-risk and common driving scenarios. The potential crash scenarios included in the VDT driving environments were determined by the National Highway Traffic Safety Administration [15,16], including intersections (3- and 4-way, stop sign, and traffic light), curved roads, rear-end events (lead car brakes suddenly), and hazard zones (construction zones, school zones, and pedestrian crosswalks). These typical driving scenarios were specified by subject matter experts from the state's licensing and motor vehicle safety body encompass a variety of settings (eg, urban and rural) and on-road elements (eg, school buses, ambulances, pedestrians, and hazards) to reproduce local driving environments. As a result, the VDT provides an opportunity for a wide variety of driving responses.

Consequently, a large quantity of applicant time series data was generated from the VDT assessment. To efficiently create a prediction (ORE pass/fail) scoring algorithm, steps must be taken to reduce the dimensionality of the data for analysis. Many traditional machine learning (ML) methods (*out-of-the-box*) require multiple domain-specific features that are well defined and created before being evaluated in an ML pipeline. Given the task of predicting the ORE outcome at the individual level, manually defining features with optimized predictive capabilities is an extremely resource-intensive task and often reliant on subject matter expertise (SME) and considerable domain knowledge.

Therefore, by leveraging data collected from the VDT pilot, this study aimed to develop and conduct an initial evaluation of a novel ML-based classifier using limited domain knowledge and minimal feature engineering to reliably predict applicant pass/fail on the ORE. Such methods, if proven useful, could be applicable to the classification of other time series data collected within the medical field and other settings.

## Methods

### Apparatus

**Figure 1:**
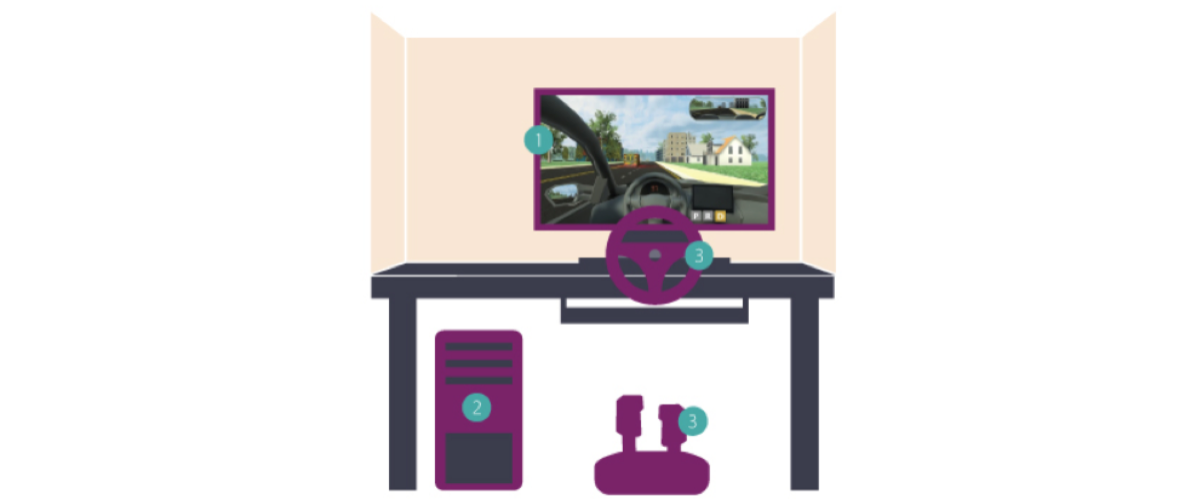
Workstation Setup: 1) Standard Monitor, 2) Standard Desktop Computer, 3) Off-the-shelf USB Steering Wheel and Pedals.

The VDT used in this pilot was delivered by Diagnostic Driving, Inc [17] in collaboration with Children’s Hospital of Philadelphia (CHOP) and designated state-employed highly experienced driving examiners (SMEs). The VDT software evolved from a prior laboratory-based SDA into a highly scalable, portable, and self-directed tool (Figure 1) that could be delivered on ubiquitous hardware without the need for additional personnel to help facilitate its delivery (eg, research assistants and administrative personnel; Multimedia Appendices 1 and 2).

### Virtual Driving Test Implementation Procedure

**Figure 2:**
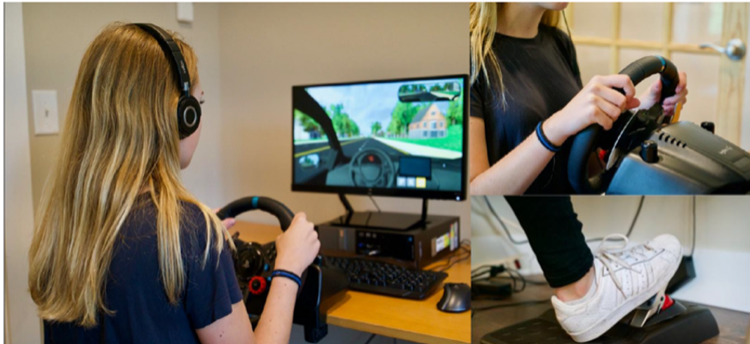
Virtual driving test workstation.

Applicants were directed to an available VDT workstation in the testing facility by state personnel, and applicants were asked to put on headphones (Multimedia Appendix 2) and enter the provided unique identification number that could be linked to their ORE results (Figure 2).

The VDT is a self-directed workflow (containing both on-screen and voice-directed instructions) that typically takes less than 15 min to complete after the applicant logs in to the VDT workstation with a unique identification number provided by state personnel. A short orientation video introduces the importance of safe driving and provides the applicant with an overview of the stages of the VDT workflow. This is followed by a simulated *practice drive,* a short orientation drive with built-in instructions to orient the applicant to all VDT controls (eg, turn signals, transmission controls, and navigation system) and to allow the applicant to test drive basic maneuvers (eg, steering, accelerating, braking, and 90° turns) on a course without additional traffic. This drive ends with a brief comprehension test that evaluates the driver applicant's ability to manage the controls and to follow basic instructions covered in it.

Next, the applicant begins the simulated *assessment drive,* a planned route through a randomly assigned environment selected from a bank of 10 possible environments (eg, *city 1, city 2, ..., city 10*), all of which contain variations of common and serious crash scenarios. In all the environments, driver applicants are never explicitly prompted to react to changing traffic conditions or traffic controllers (eg, *grant pedestrians in the crosswalk right of way* and *wait for the light to turn green*). They are, however, given navigational instructions to follow a planned route (eg, *turn left at the stop sign* and *shift into the right lane*). The assessment drive has no explicit time limit and finishes once the applicant brings his or her simulated vehicle to the end of the planned route.

On completion of the assessment drive, the applicant receives a series of 3 debriefing questions to answer on the screen (using a 5-point Likert scale: 1=strongly disagree and 5=strongly agree) to assess his or her ability to understand VDT directions and general comfort with the VDT controls.

### Dataset

We received a deidentified dataset containing individually linked VDT and ORE performance data for 4643 driver applicants from 3 licensing facilities collected between July 2017 and March 2018. These data were collected by a large Midwestern state during a pilot phase of implementing the VDT in collaboration with Diagnostic Driving, Inc, and CHOP to test its utility as a screening tool in the context of a high-demand and established state government licensing workflow. During this pilot phase, no additional research or demographic information (eg, age and sex) was collected, and all driver applicants who completed the VDT went on to complete the ORE regardless of VDT performance.

In addition, neither the driver applicant nor the licensing examiner had access to the VDT results; therefore, no bias because of VDT performance was introduced when implementing the ORE. Driver applicant ORE outcomes were linked to VDT data and entered into a deidentified dataset that was shared with the research team. CHOP’s institutional review board determined that this study does not meet the criteria for human subjects research primarily because (1) data were not obtained by the researchers through intervention or interaction with the individual and (2) no identifiable private information was included in the dataset received.

### Derivation of Sample

**Figure 3:**
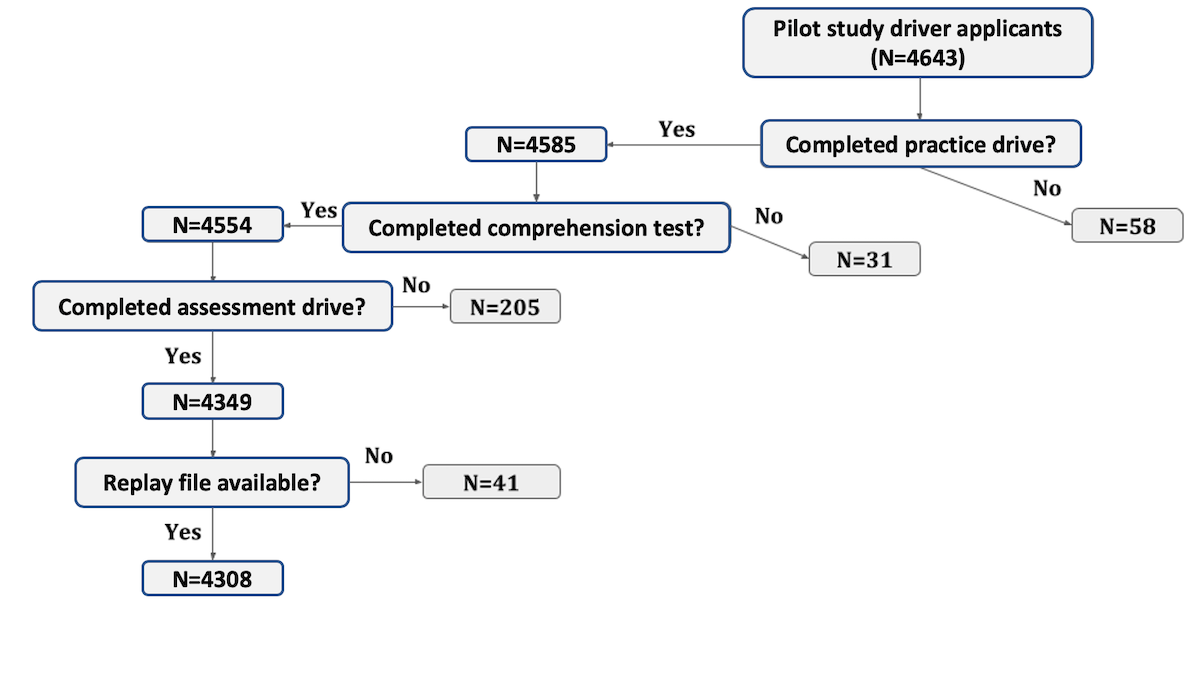
Sample derivation: data from 335 (7.2%) enrolled driver applicants were excluded from the final sample of 4308 because they either did not complete the VDT workflow or their assessment replay file was unavailable for analysis. VDT: virtual driving test.

Figure 3 shows the derivation of the sample included in the analyzable dataset. Of the 4643 driver applicants who attempted the VDT in the pilot, the vast majority completed it: 58 (1.2%) applicants did not complete the practice drive, 31 (0.7%) did not complete the comprehension test, and 205 (4.4%) did not complete the assessment drive (descriptions of comprehension and assessment drives are given in the Virtual Driving Test Implementation Procedure section). Driver applicants who did not complete the entire VDT workflow, 6.3% (294/4643) were excluded from our analysis. SMEs from the licensing facilities provided information on why some applicants could not complete the workflow, and reasons included applicant had a language barrier, applicant did not understand the instructions, applicant was frustrated with the VDT software, applicant was called for their ORE earlier than expected, applicant walked away from the VDT workstation, applicant experienced symptoms of simulator sickness, and applicant elected not to continue (<1%). In addition to not completing the entire VDT workflow, an additional 41 (0.9%) applicants who completed the workflow did not have their replay files (raw time series information) successfully uploaded to the VDT cloud server (because of internet connectivity issues). These cases were also removed from our analysis, and the final analyzable sample included 4308 (92.8%) driver applicants.

#### Data Collected: Time Series Channels

The VDT software is fully cloud based and collects raw time series–driving performance data, sampled at 60 Hz, including the driver’s vehicle position; driver inputs (ie, steering wheel, brake, and throttle); additional driver attributes (ie, velocity); and information regarding ambient vehicles, pedestrians, and environmental objects. This raw and rich representation is then downsampled (for the purpose of storage) at approximately 10 Hz with the true elapsed time between contiguous frames recorded, with the added benefit of reducing the replay file size for permanent cloud storage while retaining the faithfulness of the time series representation. As each *replay file* (the downsampled recording of a VDT assessment) is stored as a multichannel sequence of frames averaging about 8 min in length downsampled to roughly 10 Hz, it provides more than 30,000 data points of information regarding driving performance.

Each frame of the recorded time series data includes several raw values regarding the applicant’s physical interaction with the VDT workstation, including (1) the percentage of complete brake depression (with 1=full depression/stop) and throttle pedals (with 1=full depression/maximal acceleration), (2) the signed percentage of steering wheel rotations both left (negative) and right (positive) of its resting position, and (3) the position and heading of the applicant’s vehicle within the simulated environment. Also recorded in each frame is the use of turn signals and steering wheel buttons that allow the driver to scan left or right to look for oncoming traffic.

The *lane offset* (sometimes referred to as lane position) [18] of the applicant’s vehicle within its lane on the simulated road is computed for each frame and recorded as one of the time series channels. These channels comprise each applicant’s VDT assessment recording and are used as the basis for feature set construction to represent driving performance.

#### Outcome Variable

For all driver applicants who completed the VDT, the result of the ORE is provided in a numeric form (score), where a higher score indicates a more severe accumulation of infractions cited by the examiner during their ORE. According to the state licensing agency’s ORE scoring protocol, any driver with a score greater than or equal to 26 fails the ORE and any driver with a score less than 26 passes the ORE (a score of 0 indicates a perfect score). These data (both numerical and dichotomous pass/fail representations) for each applicant are linked to the corresponding VDT record and added to the deidentified dataset. For the purposes of internal analysis, we define the *gray zone* of ORE scores between 20 and 35, where drivers either barely pass or accumulate just enough infractions to fail the ORE.

### Analytical Procedure

To maximize our ability to reliably predict an applicant’s likelihood of failing the ORE, we evaluated 2 alternative ways to represent the time series data. The first, standard approach (which we call the *variables* feature set) involves the reduction of the driver applicant's input time series data into manually created variables that represent driver behavior These variables further represent both continuous features (eg, velocity) and count/dichotomous data (eg, crash). The second method is novel and involves viewing the environments as a series of predefined *event zones* to which the applicant is exposed (eg, a crosswalk, an intersection, or a school zone). The corresponding applicant’s input time series data (how the applicant responds to each of these event zones) are then partitioned according to these event zones. The range of applicant behaviors (combinations of steering, brake, and throttle on the simulated roadway) is then clustered for each segment without any preprocessing or manual reduction of the data. This *time series clustering* feature set has no shared features with the *variables* feature set.

**Figure 4:**
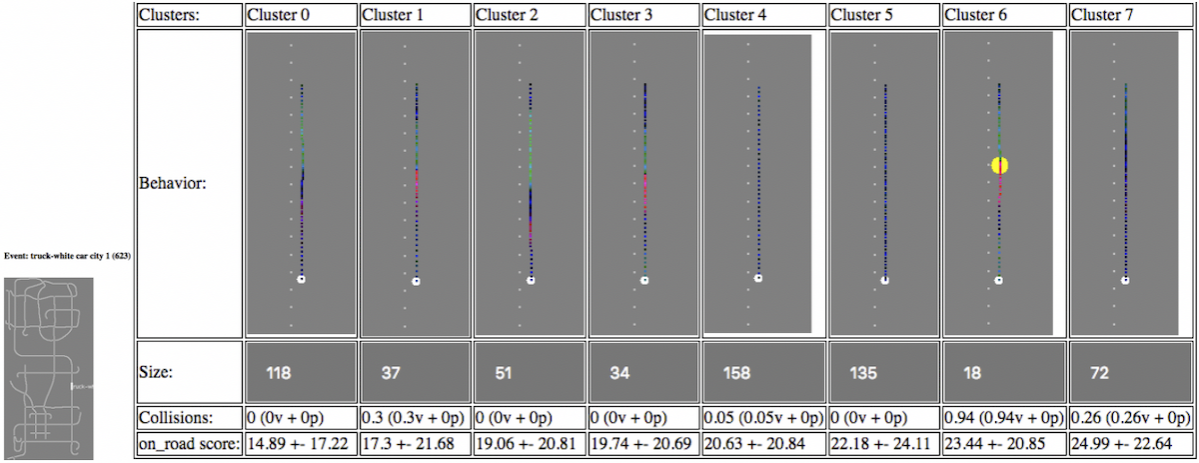
Time Series Clustering for highlighted event zone with cluster centers representing prototypical driving behaviors in that zone.

To build classifiers of driver performance, we constructed these 2 feature sets as input for supervised ML algorithms (Figure 4).

#### Standard Method for Data Reduction: Variables Feature Set

For every simulated drive, there are events corresponding to common and serious potential crash scenarios. Applicants’ responses that have been tracked and tabulated in real time from within the VDT-simulated environment (eg, reduction of speed entering a construction zone) are augmented with manually created variables from the stored data, which indicate aberrant or hazardous driving behaviors (eg, number of instances applicant ran a red traffic light).

A summary of speed management within a sample is represented by the global maximum, mean, and median of the applicant vehicle velocities as well as the ratio of the applicant vehicle’s velocity to the posted speed limit recorded. The same statistics on velocity and speed ratio are computed for the following event zones: crosswalks, school zones, construction zones, playground zones, and banking curves. To flush out adherence to the posted speed limit, the global percentages of each sample driven more than 10, 15, and 20 mph above and below the posted speed limit are used as potential predictors.

These values, along with other computed driving performance metrics, result in a representation of each sample as a vector of 67 engineered features plus the *outcome* variable (ORE pass/fail). With this representation of the tabulated data, supervised learning methods are deployed using standard 10-fold cross-validation; we examined the resulting confusion matrices. To evaluate the predictive power of this representation, we examined the resulting confusion matrices.

#### Novel Method for Automated Data Reduction: Time Series Clustering Feature Set

Each applicant’s stored time series data were truncated to begin at the first downsampled frame with a simulated speed above 0.5 m per second to align replay files via a common starting condition. As assessment drives are of nontrivial length (on average, approximately 8 min in duration), relying on global measurements to compare driving behaviors may wash out highly predictive features from smaller segments of the assessment’s planned route.

The *time series clustering* representation attempts to derive new features from sections of the drives that are designed to elicit specialized behaviors in response to environmental or traffic conditions. Unlike the standard variables approach, this new approach derives features automatically without the need for manual data reduction. Time series samples are grouped by the simulated environment to which the applicant is assigned, where each environment comprises a sequence of stationary event zones defined by *entry* and *exit* *waypoints*. Given a sample that drives through a particular event zone, a *subinterval* of the time series is extracted that corresponds to the vehicle traveling between the zone’s *entry* and *exit* waypoints. This subinterval is grouped in a set with all other subintervals from the drives that pass through the designated event zone.

The purpose of clustering is to reveal *prototypical behaviors* so that drivers can be categorized within each event zone (as defined by their lane position, throttle, brake, accelerator, and steering wheel usage in each frame). For example, at a crosswalk, there might be a cluster for those who slow to a stop and another for those who speed through the crosswalk without slowing. The variance in behaviors should be represented in the cluster centers, where all sample subintervals assigned to a given cluster have been determined to be most similar to the cluster center’s *prototypical behavior*.

Speed and traffic conditions determine each driver’s subinterval length in an event zone. Thus, to compare subintervals of unequal lengths, dynamic time warping [19,20] is used as the (dis)similarity measure, where the time steps of pairs of subintervals are aligned to minimize the time-warped aggregate difference between them. *k*-Medoids [21], a derivative of *k-means*, is used to pigeonhole the n samples into k clusters (*k=*8 in all the reported experiments) by electing k initial subintervals 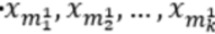 to act as *medoids*, cluster centers representing prototypical behaviors. A dissimilarity matrix 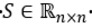 is constructed, where each cell 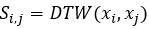 compares subintervals χ*_i_* and 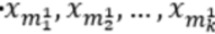 numerically. The subinterval χ*_i_* is assigned to cluster 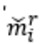, with the most similar medoid at round *r* of iteration. The algorithm elects new medoids 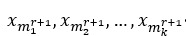 in each round by identifying the candidate subinterval within each cluster 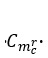 that maximizes the aggregate similarity (ie, minimizes dissimilarity) between it and all other cluster members.

At every round of clustering the *inertia*, the sum of squared residual dissimilarities between subintervals and their assigned medoids is computed. If the newly elected medoids reduce inertia from the round before, the cycle repeats itself, although we imposed a limitation of 100 repetitions. Once *k-Medoids* converge on a clustering that cannot be improved upon, the dissimilarities between each sample subinterval χ*_i_* to medoids 
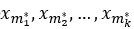
 are arranged as a feature vector.

This method of reporting the medoid dissimilarities can be thought of as *soft clustering*, where we report the relative *membership* of each subinterval to all clusters. As the initial medoids are randomly selected, 20 initializations of clustering are run for each event zone, using the medoids that minimize the overall inertia to represent the range of prototypical behaviors for the given event zone. This is done to select the most *compact* clusters where samples grouped together minimize aggregate dissimilarity from the medoids, distilling the prototypical behaviors that best represent the range of behaviors observed.

Illustrated on the far-left side of Multimedia Appendix 3 is a bird’s-eye view of 1 of the 10 simulated environments’ track, with a specific event zone highlighted (eg, *prescripted traffic interaction and avoid collision response to white car ahead suddenly stops in roadway*). Depicted on the right side of Multimedia Appendix 3 is the range of prototypical behaviors exhibited by the identified medoids while navigating through the indicated event zone.Beneath the depictions of each prototypical behavior is recorded the number of subintervals in its cluster, the percentage of those subintervals that had collisions in the defined event zone (both with vehicles and pedestrians), as well as the means and standard deviations of ORE scores from all cluster members.

With 166 different event zones in our dataset, each sample was converted to a concatenated feature vector of 1328 medoid dissimilarity features (*k×*166). For samples that never encounter a given event zone, the dissimilarities to the generated medoids are left as *k* blank entries. With the data table of medoid dissimilarities constructed as features and every sample having an *actual* pass/fail *outcome label*, supervised learning is trained and tested on the dataset using standard leave-one-out 10-fold cross-validation.

### Supervised Learning Methods

Supervised learning methods evaluated features (both observed and derived) to establish their ability to predict a given driver applicant’s *outcome label* (ORE pass/fail). Specifically, all methods were evaluated using a standard 10-fold cross-validation in an attempt to minimize the overfitting of any model. We evaluated 2 classification methods within the Waikato Environment for Knowledge Analysis [22,23]: logistic regression and support vector machines (SVMs).

#### Logistic Regression

Logistic regression ML algorithms classify a given sample based on computing its probability of belonging to one of the binary classes defined by the pass/fail *outcome label*. Given the predicted probability that a sample has either passed or failed, a decision rule is used to classify the sample:


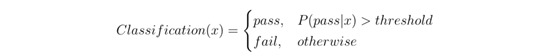


In this example, the outcome label is based on actual ORE pass/fail, and the probability threshold used in the decision rule is derived from 0.0 to 1.0, with a step size of 0.01, to explore the threshold parameter’s effect on performance metrics.

#### Support Vector Machines

SVMs attempt to compute the optimal decision surface for partitioning datasets along with binary class values (in our case, *pass* and *fail*) by maximizing the margin of separation between samples known to be members of different classes [24]. The core assumption of an SVM model is that there exists a transformed space in which the dataset may be linearly partitioned via a hyperplane. As it is computationally costly to transform every sample, a *kernel function* was used to relate pairs of samples in the transformed space.

For all results using SVM classification, we elected to use the *radial basis function* (RBF) as our kernel because our initial experimentation suggested that our feature values resemble Gaussian distributions. A coarse grid search was used to narrow the ranges of parameters considered for building an effective classifier [25]. SVMs for binary classification are primarily configured using 2 different values: a *cost* parameter (which determines the degree of influence of data points far from the decision surface in the algorithm) and a *gamma* parameter (which controls the variance of the Gaussian functions that make up the RBF kernel). We dyadically iterated the *cost* from 2^−5^ to 2^−15^ by doubling. To perform a grid search in a 2-dimensional parameter space, we also dyadically iterated the *gamma* parameter from 2^−10^ to 2^6^ by doubling.

### Evaluation Metrics

As previously described, the primary goal of the VDT pilot was to reliably assess applicant preparedness before taking the ORE, thereby maximizing the safety of driving examiners and providing an opportunity for prepared applicants to take the ORE. The evaluation metrics described in the following sections were used to evaluate a given model’s predictive ability while addressing the goals of the VDT pilot.

#### Confusion Matrix and Evaluation Metrics

To predict a binary outcome of the ORE with confidence, a confusion matrix (see Table 1) for each classification model was generated to evaluate the predictive ability of our classifiers.

Our evaluation metrics included accuracy, algorithm fail rate, false alarm rate, ratio of false alarm, and risk ratio.

*Accuracy* was defined as the ratio of the total number of correctly classified cases to the total sample population:


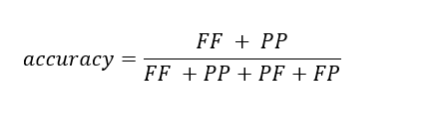


Algorithm fail rate was defined as the percentage of total cases the classifier predicted as fail:


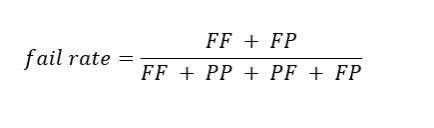


False alarm rate was defined as the percentage of total cases the classifier misclassified as fail:


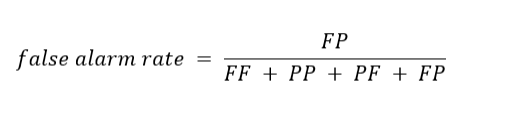


The *ratio of false alarms* was defined as the ratio of cases of misclassified *fail* to the total cases where the classifier predicted *fail*:


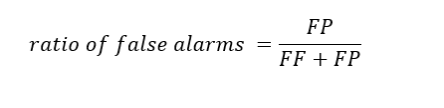


The *risk ratio* was defined as the relative risk (RR) of failing the ORE when the classifier predicted *fail* versus predicting *pass*. The 95% CIs were also calculated for risk ratios:


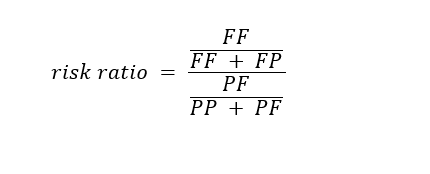


SMEs within the licensing agency recommended that a satisfactory classifier would consist of a model that maximized both accuracy and risk ratio while minimizing the false alarm rate (applicants who failed the VDT but went on to pass their ORE).

**Table 1:** A confusion matrix, showing the 4 quadrants: FF (fail-fail), FP (fail-pass), PF (pass-fail), and PP (pass-pass).

Confusion matrix	Fail ORE^a^	Pass ORE
Fail VDT^b^	FF	FP
Pass VDT	PF	PP

^a^ORE: on-road examination.

^b^VDT: virtual driving test.

#### Fitness of Models

In addition to the evaluation metrics previously described, we also evaluated model fitness using *receiver operator characteristic* (ROC) curves to compare our predictions against a random binary classification. An ROC curve illustrates a model’s performance using different parameterizations. Specifically, it plots the model’s *true positive rate* (TPR) to its *false positive rate* (FPR). For our data, TPR (also known as sensitivity) is the ratio of driver applicants who are correctly predicted to fail the ORE to the overall population of applicants who went on to fail the ORE:


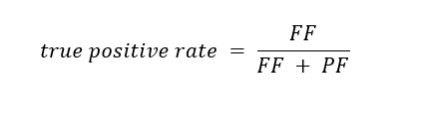


FPR is the ratio of driver applicants incorrectly predicted to fail the ORE to the overall population of applicants who went on to pass the ORE:


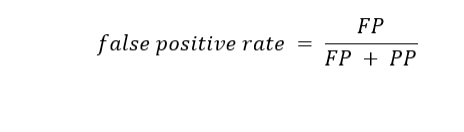


Classifiers that have equal TPR and FPR do not perform well, as there is less certainty that the classification of any particular sample is reliable. ROC curves that deviate significantly from the line TPR=FPR represent classifiers that perform better at distinguishing the 2 classes. A perfect classifier would have a TPR of 1.0 and an FPR of 0.0, where a random binary classifier would have these 2 values be the same. A common metric used to summarize the relationship between TPR and FPR over a range of model parameterizations is the *area under the curve* (AUC). AUC is the numerical integral of the ROC curve (FPR is the independent variable and TPR is the dependent variable), where a higher percentage of the total area coverable (TPR of 1.0 for all values of FPR) should indicate models that are more fit to reliably predict a class value (eg, ORE outcome) [26].

## Results

### Classifier Results

We summarize the classifier results with the evaluation metrics previously described in Table 2. In this table, results from 4 classifiers were reported: feature sets generated from 2 methods (*variables* and the novel approach) and *time series clustering*, used in 2 ML methods (logistic regression and SVM). Overall, the results from the *time series clustering* approach provided similar results to the *variables* approach. More specifically, logistic regression using the time series clustering approach to generate features produced the best classifier based on our evaluation metrics (highest accuracy: 76.2%; highest risk ratio: 3.07; 95% CI 2.75-3.43). In addition, this classifier minimizes the ratio of false alarms compared with the others (27.2%).

**Table 2:** Summary results of the evaluation metrics obtained from 4 classifiers: variables+logistic regression, variables+support vector machine, time series clustering+logistic regression, and series time clustering+support vector machine.

Classifier results	Standard method (variables)		Novel method (time series clustering)	
	Logistic regression	SVM^a^	Logistic regression	SVM
Accuracy, %	76.1	75.4	76.2	74.9
Fail rate, %	6.6	3.4	3.5	3.6
False alarm rate, %	2.6	1.3	1.0	1.7
Ratio of false alarms, %	38.5	37.7	27.2	45.9
Relative risk (95% CI)	2.684 (2.409-2.991)	2.581 (2.250-2.961)	3.071 (2.747-3.434)	2.223 (1.906-2.592)
True positive rate, %	15.9	8.3	10.0	7.8
False positive rate, %	3.4	1.7	1.3	2.2

^a^SVM: support vector machine.

### Logistic Regression

**Figure 5:**
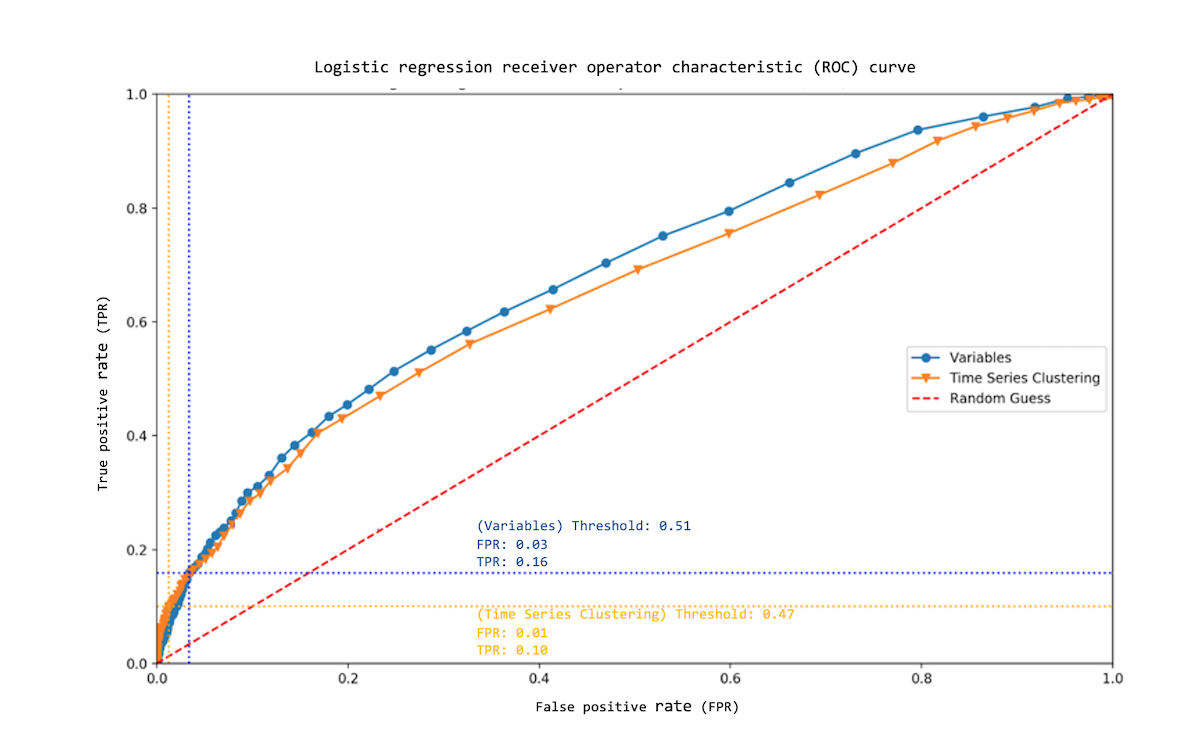
Receiver operator characteristic curves for logistic regression using the variables and time series clustering feature sets for iterated logistic cutoff threshold values. Points in the bottom left represent models with the lowest thresholds (more people pass the virtual driving test), whereas points in the top right represent models with the highest thresholds (fewer people pass the virtual driving test).

Figure 5 highlights the results for logistic regression with the highest observed classification accuracies for both representations (*variables* and *times series clustering*). The most promising parameterization of logistic regression using the *variables* representation as input for the VDT has an FPR of 3.4% and a TPR of 15.9%, whereas the most promising parameterization of logistic regression using the *time series clustering* representation as input for the VDT has an FPR of 1.3% and a TPR of 10.0%. Moreover, we observed that the *time series clustering* representation yielded a VDT classifier with a smaller ratio of false alarms (27.2%) than the most accurate VDT classifier using the *variables* representation (38.5%).

The threshold value yielding the model with the highest classification accuracy is highlighted in Figure 5. The highlighted parameterizations (yielding the highest classification accuracy, marked by the vertical dotted lines in the figure) demonstrate VDTs that show predictive promise, although there is room for improvement. Using logistic regression with different threshold values, the AUC for the *variables* feature set’s ROC curve is 0.682, whereas the AUC for the *time series clustering* feature set’s ROC curve is 0.656.

As seen in Figure 6 of Multimedia Appendix 4, logistic regression using the *variables* representation of the dataset produces a classifier that fails 6.9% of those taking the VDT, where 39% of those predicted to fail the ORE actually went on to pass (ratio of false alarms). The false alarms represent only 3% of all drivers, with 37% of the false alarms having an ORE score between 20 and 25, meaning these drivers nearly failed the ORE. Within the group of drivers misclassified (24% of all driver applicants), 30% of samples have an ORE score in the *gray zone* (20-35, with a score of ≤25 resulting in a pass). With this model, drivers failed by the VDT are 2.7 times more likely to fail the ORE (RR=2.68; 95% CI 2.41-2.97).

Using logistic regression, the *time series clustering* representation of the dataset produces a classifier that fails 4% of those taking the VDT, with a ratio of false alarms of 27%. Only 1% of all drivers are false alarms, where 25% of false alarms nearly fail the ORE with scores between 20 and 25. For all misclassified drivers (24% of all driver applicants), 25% of the samples had an ORE score in the *gray zone.* Using this model, drivers failed by the VDT are more than 3 times more likely to fail the ORE (RR=3.07; 95% CI 2.75-3.43). Overall, the *time series clustering* representation used as input for the logistic regression algorithm produces a more lax classifier that fails approximately half as many drivers as using the *variables* representation as input. However, when predicting failure on the ORE, logistic regression using the *time series clustering* representation is more often correct than with the *variables* representation (72% correct predictions of *fail* versus 62%).

### Support Vector Machines

As Figure 7 in Multimedia Appendix 5 shows, the most accurate parameterization of SVM using the *variables* representation as input for the VDT has an FPR of 1.7% and a TPR of 8.2%, whereas the most promising parameterization of SVM using the *time series clustering* representation as input for the VDT has an FPR of 2.2% and a TPR of 7.8%. Moreover, we observed that the *time series clustering* representation yielded a VDT with a larger ratio of false alarms (45%) than the most accurate VDT using the *variables* representation (38%). 

Loose grid search of SVM’s 2 parameters reinforces the notion that the 2 classes of ORE pass/fail are highly comingled within both feature spaces. Figure 8 in Multimedia Appendix 6 contains surface plots for both feature sets showing the interpolated landscapes of risk ratio and accuracy (vertical axis) over the SVM gamma and C (horizontal/lateral axes) parameters sampled in bivariate grid search.

The SVM parameterization with the highest observed classification accuracy using the *variables* representation of the dataset produces a classifier that fails 3.5% of those taking the VDT, with 38% of those predicted to fail the ORE being false alarms. The false alarms comprise 1.4% of all drivers, with 33% of the false alarms having nearly failed the ORE, where scores between 20 and 25 indicate nearly failing the ORE. Of the driver applicants misclassified (24% of all driver applicants), 26% of samples had an ORE score in the *gray zone* (20-35, with a score of ≤25 resulting in a pass). With this model, drivers failed by the VDT are 2.6 times more likely to fail the ORE, corresponding to the plateau of risk ratios illustrated in Figure 8 of Multimedia Appendix 6.

Using the *time series clustering* feature set, the SVM parameterization yielding the highest classification accuracy failed 3.6% of people taking the VDT, where 45% of those predicted to fail the ORE are false alarms. With 1.6% of all driver applicants being a false alarm, 13% of the false alarms nearly failed the ORE with scores between 20 and 25. This VDT misclassifies 25% of all driver applicants, where 26% of these drivers have an ORE score in the *gray zone*. With this model, drivers failed by the VDT are 2.3 times more likely to fail the ORE.

## Discussion

### Principal Findings

This study specifically aimed to evaluate a novel method for automatically classifying user-generated time series data versus a traditional and resource-intensive approach (classification by manually creating features [variables] using domain knowledge and SME). When compared with the standard method of feature selection (using manually created variables), the clustering method demonstrated a similar to higher accuracy in predicting ORE results. More specifically, it outperformed the standard method by accurately identifying the most underprepared applicants (those applicants who failed the ORE).

Although time series clustering is not a completely new idea, our particular application using a two-stage analysis of critical subintervals (our event zones) is novel. Moreover, our application does not suffer from the known problem of *sliding-window partitioning* *then cluster* methods, which have been shown to be inconsistent in producing meaningful predictions [27]. In this study, we leveraged contextual similarities in scripted scenarios written to elicit specialized driving behavior in response to changes in the environment along the route, including ambient traffic [28]. Clustering the behaviors exhibited in these event zones leads to highly correlated clusters [29] that are quantifiably useful for the task of predicting ORE pass/fail.

The clustering is useful beyond simple binary prediction: Knowing how the data break down into groups allowed us to formulate a profile made of *prototypical behavior*, which represents the traits exhibited by the cluster members [30]. Only a small portion of the sample (433 of the 4308 drivers) had an ORE score greater than 45 (scores <26 result in passing). As we dealt with a heavily weighted distribution of ORE pass/fail (roughly 3 to 1), we carefully scrutinized the composition of the clusters formed; we also focused on identifying dangerous drivers (higher accumulation of critical driving errors) instead of optimizing overall predictive accuracy for ORE pass/fail.

Although the clusters did not cleanly separate all those who failed from those who passed, the features generated and then classified were able to identify outliers in the data rather easily. In many cases, these outliers presented aberrant driving behaviors, such as collisions with vehicles, collisions with pedestrians, and driving off the road. As it is meant to be a prescreening test, the purpose of VDT was never to identify all those who would go on to fail the ORE but rather to isolate the driver applicants who present a danger to themselves and others on the road. Operationally, the VDT should not restrict eligible driver applicants from taking the ORE; with that in mind, our novel approach shows promise in confidently classifying truly unprepared driver applicants. As a result, we considered evaluation metrics such as risk ratio instead of accuracy to keep state ORE examiners out of harm’s way.

### Limitations

This study had some limitations. First, the *variables* approach that we used did not represent the entire domain set of VDT performance metrics that could be derived and used. Many of the initial variables focused heavily on global measures (eg, measures across the entire simulated drive) rather than focusing on event-specific measures (eg, performance on curves and intersections). Owing to this, our analyses may not have taken into account the full variation in differential VDT performance (as we would expect that event-specific measures would be able to further explain this variability).

Second, the sample size available precluded model formulation specific to the simulated environment (eg, 10 separate models). Although assignment to clusters was performed for each of the 10 unique environments, only 1 dataset determined model formulation: each driver applicant record included a series of cluster assignments and their corresponding ORE score. Future research with larger samples will account for the different simulated environments and evaluate them individually.

Finally, because of restrictions placed on implementing the VDT in a busy licensing workflow, our limited dataset did not include demographic information and confounding variables collected for this sample. Age and sex are known risk factors for crashes among novice drivers [31] and may explain some variability in performances among new drivers seeking a driver’s license.

### Conclusions and Future Directions

This study provided initial evidence that the *time series clustering* feature set when used as input produced classifiers that performed just as well and, in some cases, better than those using the traditional *variables* approach. Future work will evaluate this method with larger sample sizes and potentially integrate it with other known methods to develop an optimized classifier. We also plan to explore more sophisticated variants of this approach, specifically expanding on a predefined number of clusters and using approaches such as the *silhouette method* [32] to determine an appropriate number of clusters for grouping behaviors exhibited by driver applicants in each event zone. Alternatively, we may attempt to maximize feature variance between clusters using a secondary *analysis of variance* clustering refinement procedure [33]. For the purpose of improved differential diagnostic capabilities in time series classification tasks, we intend to pursue several avenues of research that demonstrate potential for producing distinct prototypical user behaviors.

## Appendix

Multimedia Appendix 1 Workstation Setup: 1) Standard Dell Monitor, 2) Standard Dell PC, 3) Logitech G29 USB Steering Wheel and Pedals.

Multimedia Appendix 2 Virtual driving test workstation.

Multimedia Appendix 3 Time Series Clustering for highlighted event zone with cluster centers representing prototypical driving behaviors in that zone. In the eight sub-plots, “prototypical behaviors” represent the derived cluster medoids for a specific event zone. Plots with more densely congregated and colorized pixels indicate the driver was moving more slowly through the given event zone than a plot with sparser, colorized pixels. The white dashes indicate the road median; time series subintervals begin at the white circle and the position of the driver applicant in each frame is a colorized pixel. Red corresponds to usage of the brakes, green corresponds to the throttle, light blue represents steering, and the spacing of these pixels are determined by the vehicle’s speed in the given zone. Collisions with other vehicle or pedestrians are indicated by red circles, collisions with static environmental objects are indicated by yellow circles. Displayed underneath are the means and standard deviations of actual ORE scores of samples in each cluster. In addition, displayed is the number of samples in each cluster and the average number of collisions with vehicles and pedestrians in a given cluster.

Multimedia Appendix 4 Performance Metrics and Confidence Intervals for Logistic Regression using “Variables” and “Time Series Clustering” representations as feature sets for iterated logistic cut-off threshold values.

Multimedia Appendix 5 The most successful SVM parameterizations for both feature sets learn to distinguish data which is largely homogeneous with high “Cost” penalties for misclassification errors. The optimal trade-off observed between TPR and FPR occurs with a fail rate of approximately 3.5%, about 40% of samples failed are false alarms.

Multimedia Appendix 6 We consistently observed more SVM parameterizations that better predicted ORE pass/fail using the “Variables” feature set as input than for “Time Series Clustering”. Zero is imputed for undefined Risk Ratios in these plots, such results originate from VDTs with parameterizations that fail no one.

## Abbreviations

AUC: area under the curve
CHOP: Children’s Hospital of Philadelphia
FPR: false positive rate
ML: machine learning
MVC: motor vehicle crash
ORE: on-road examination
Penn: University of Pennsylvania
RBF: radial basis function
ROC: receiver operator characteristic
RR: relative risk
SDA: simulated driving assessment
SME: subject matter expertise
SVM: support vector machine
TPR: true positive rate
VDT: virtual driving test
