# Supplementary material for: Simulator Pre-Screening of Underprepared Drivers Prior to Licensing On-Road Examination: Clustering of Virtual Driving Test Time Series Data
Source: J Med Internet Res. 2020 Jun 18;22(6):e13995. doi: 10.2196/13995 (PMC7333075; doi:10.2196/13995)
Supplement: Multimedia Appendix 1 [file jmir_v22i6e13995_app1.docx]

**Multimedia Appendix 1**


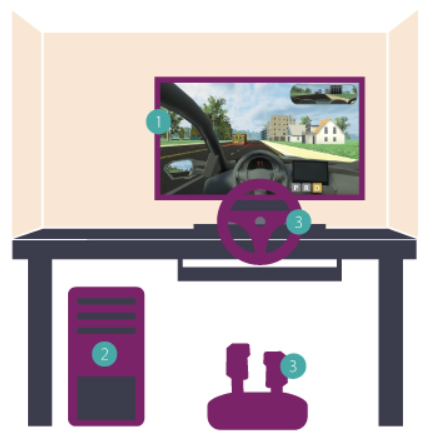


Figure 2: *Workstation Setup: 1) Standard Dell Monitor, 2) Standard Dell PC,
3) Logitech G29 USB Steering Wheel and Pedals.*
