# Supplementary figures and images for: Simulator Pre-Screening of Underprepared Drivers Prior to Licensing On-Road Examination: Clustering of Virtual Driving Test Time Series Data
Source: J Med Internet Res. 2020 Jun 18;22(6):e13995. doi: 10.2196/13995 (PMC7333075; doi:10.2196/13995)

**Multimedia Appendix 2**


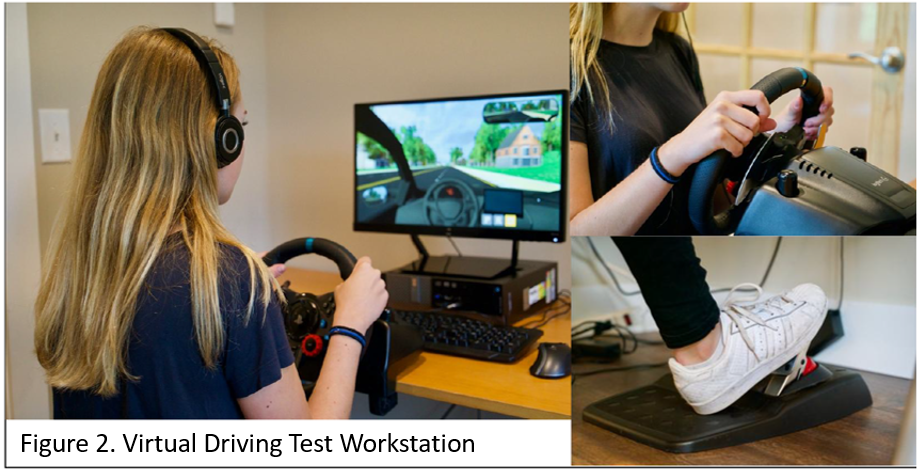


Figure 3: *Virtual driving test workstation.*

Supplement: Multimedia Appendix 2 [file jmir_v22i6e13995_app2.docx]
