# Supplementary material for: Simulator Pre-Screening of Underprepared Drivers Prior to Licensing On-Road Examination: Clustering of Virtual Driving Test Time Series Data
Source: J Med Internet Res. 2020 Jun 18;22(6):e13995. doi: 10.2196/13995 (PMC7333075; doi:10.2196/13995)
Supplement: Multimedia Appendix 3 [file jmir_v22i6e13995_app3.docx]

**Multimedia Appendix 3**

**
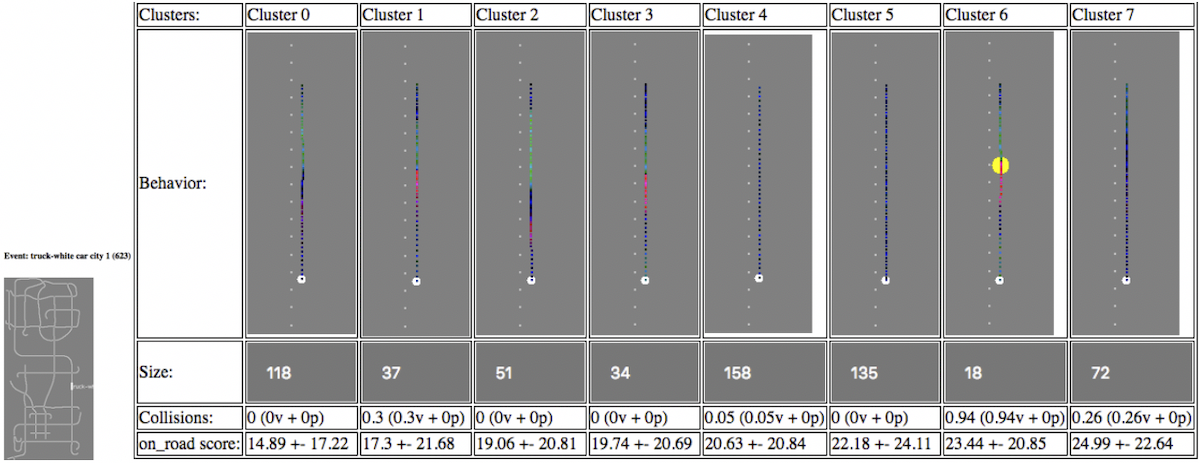
**

Figure 4: *Time Series Clustering for highlighted event zone with cluster centers representing prototypical driving behaviors in that zone. In the eight sub-plots, “prototypical behaviors” represent the derived cluster medoids for a specific event zone. Plots with more densely congregated and colorized pixels indicate the driver was moving more slowly through the given event zone than a plot with sparser, colorized pixels. The white dashes indicate the road median; time series subintervals begin at the white circle and the position of the driver applicant in each frame is a colorized pixel. Red corresponds to usage of the brakes, green corresponds to the throttle, light blue represents steering, and the spacing of these pixels are determined by the vehicle’s speed in the given zone. Collisions with other vehicle or pedestrians are indicated by red circles, collisions with static environmental objects are indicated by yellow circles. Displayed underneath are the means and standard deviations of actual ORE scores of samples in each cluster. In addition, displayed is the number of samples in each cluster and the average number of collisions with vehicles and pedestrians in a given cluster.*
