# Supplementary material for: Simulator Pre-Screening of Underprepared Drivers Prior to Licensing On-Road Examination: Clustering of Virtual Driving Test Time Series Data
Source: J Med Internet Res. 2020 Jun 18;22(6):e13995. doi: 10.2196/13995 (PMC7333075; doi:10.2196/13995)
Supplement: Multimedia Appendix 4 [file jmir_v22i6e13995_app4.docx]

**Multimedia Appendix 4**


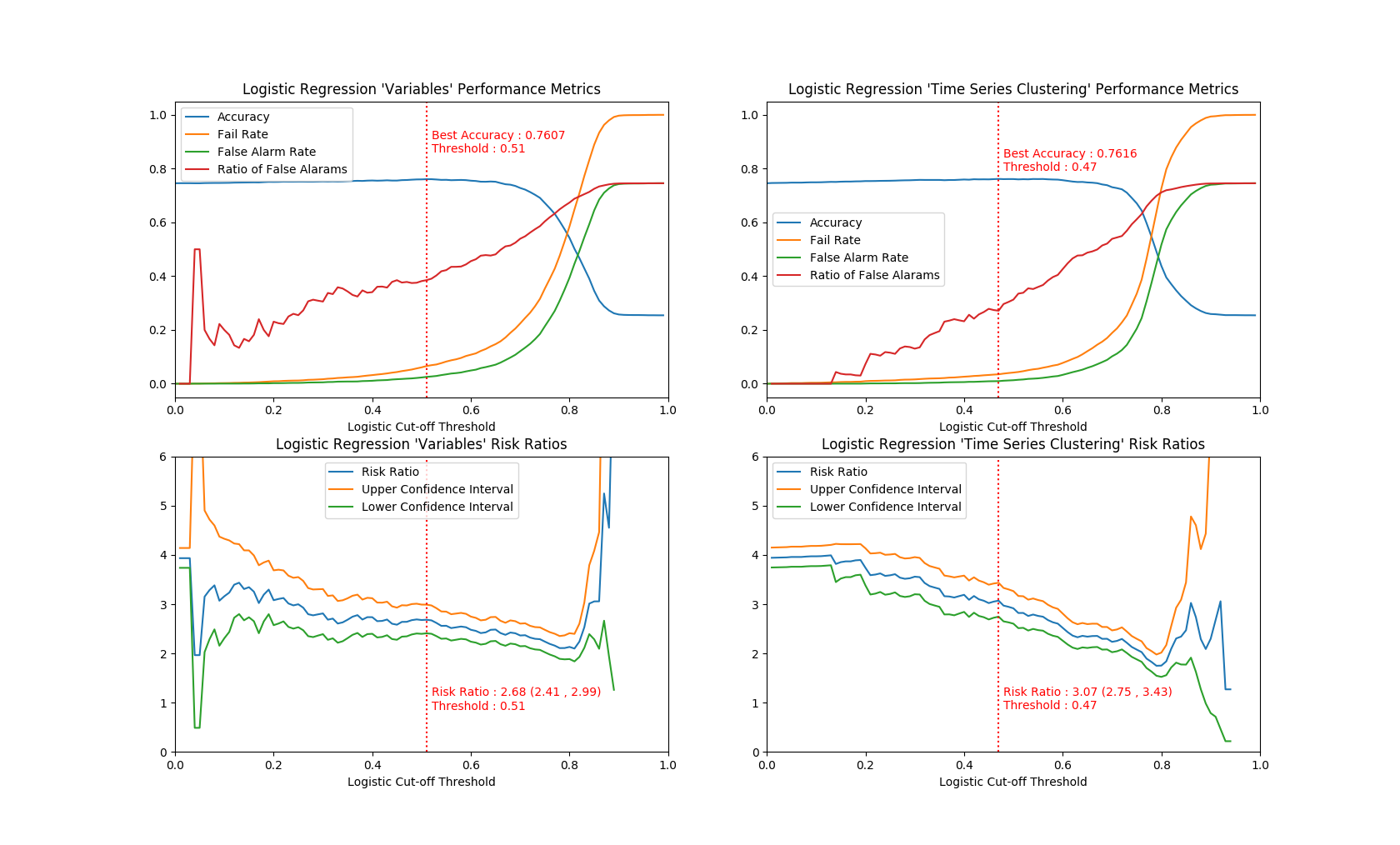


Figure 6: *Performance Metrics and Confidence Intervals for Logistic Regression using “Variables” and “Time Series Clustering” representations as feature sets for iterated logistic cut-off threshold values.*
