# Supplementary material for: Simulator Pre-Screening of Underprepared Drivers Prior to Licensing On-Road Examination: Clustering of Virtual Driving Test Time Series Data
Source: J Med Internet Res. 2020 Jun 18;22(6):e13995. doi: 10.2196/13995 (PMC7333075; doi:10.2196/13995)
Supplement: Multimedia Appendix 5 [file jmir_v22i6e13995_app5.docx]

**Multimedia Appendix 5**

*
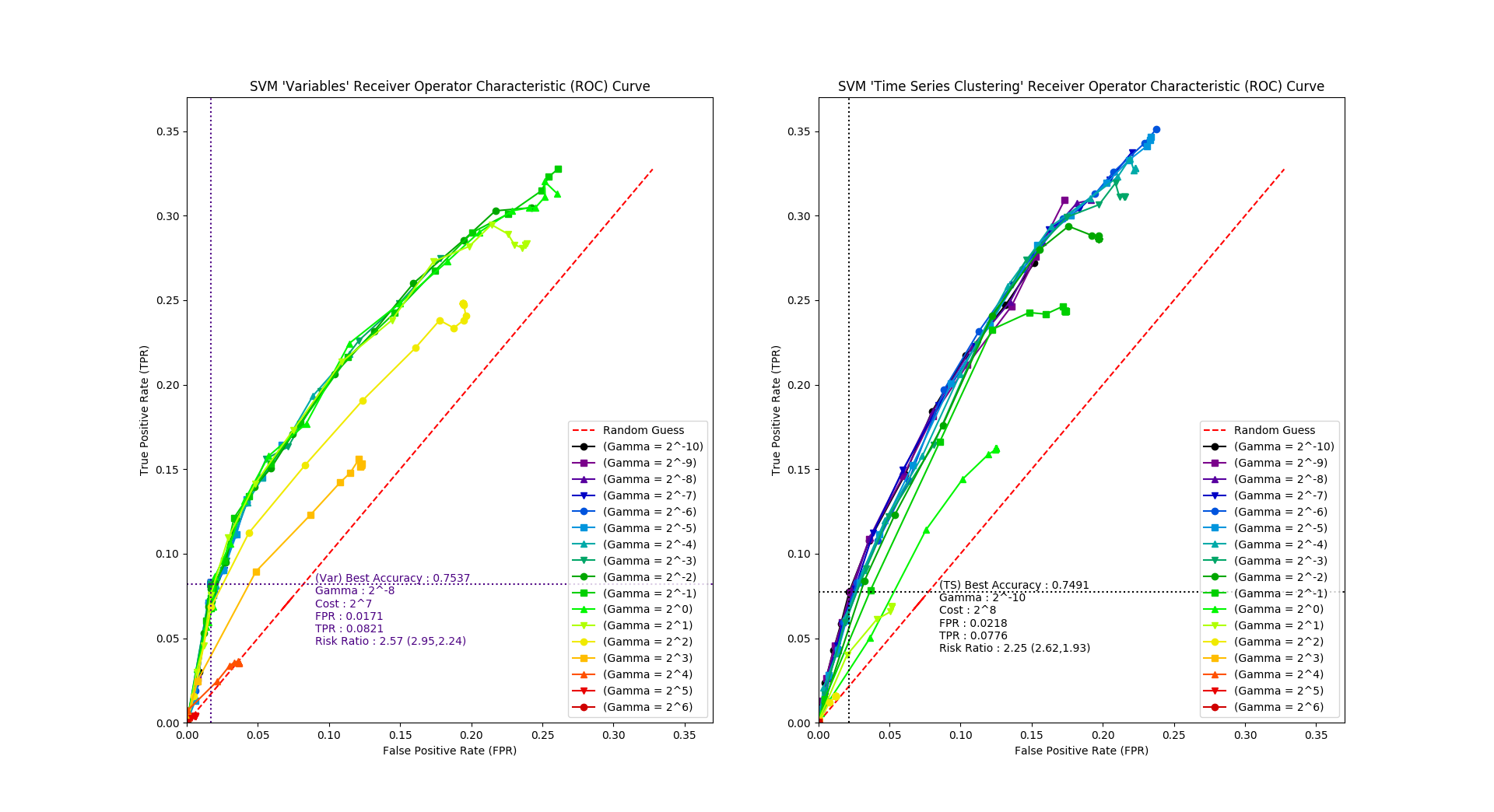
*

Figure 7: *The most successful SVM parameterizations for both feature sets learn to distinguish data which is largely homogeneous with high “Cost” penalties for misclassification errors. The optimal trade-off observed between TPR and FPR occurs with a fail rate of approximately 3.5%, about 40% of samples failed are false alarms.*
