# Supplementary material for: Simulator Pre-Screening of Underprepared Drivers Prior to Licensing On-Road Examination: Clustering of Virtual Driving Test Time Series Data
Source: J Med Internet Res. 2020 Jun 18;22(6):e13995. doi: 10.2196/13995 (PMC7333075; doi:10.2196/13995)
Supplement: Multimedia Appendix 6 [file jmir_v22i6e13995_app6.docx]

**Multimedia Appendix 6**


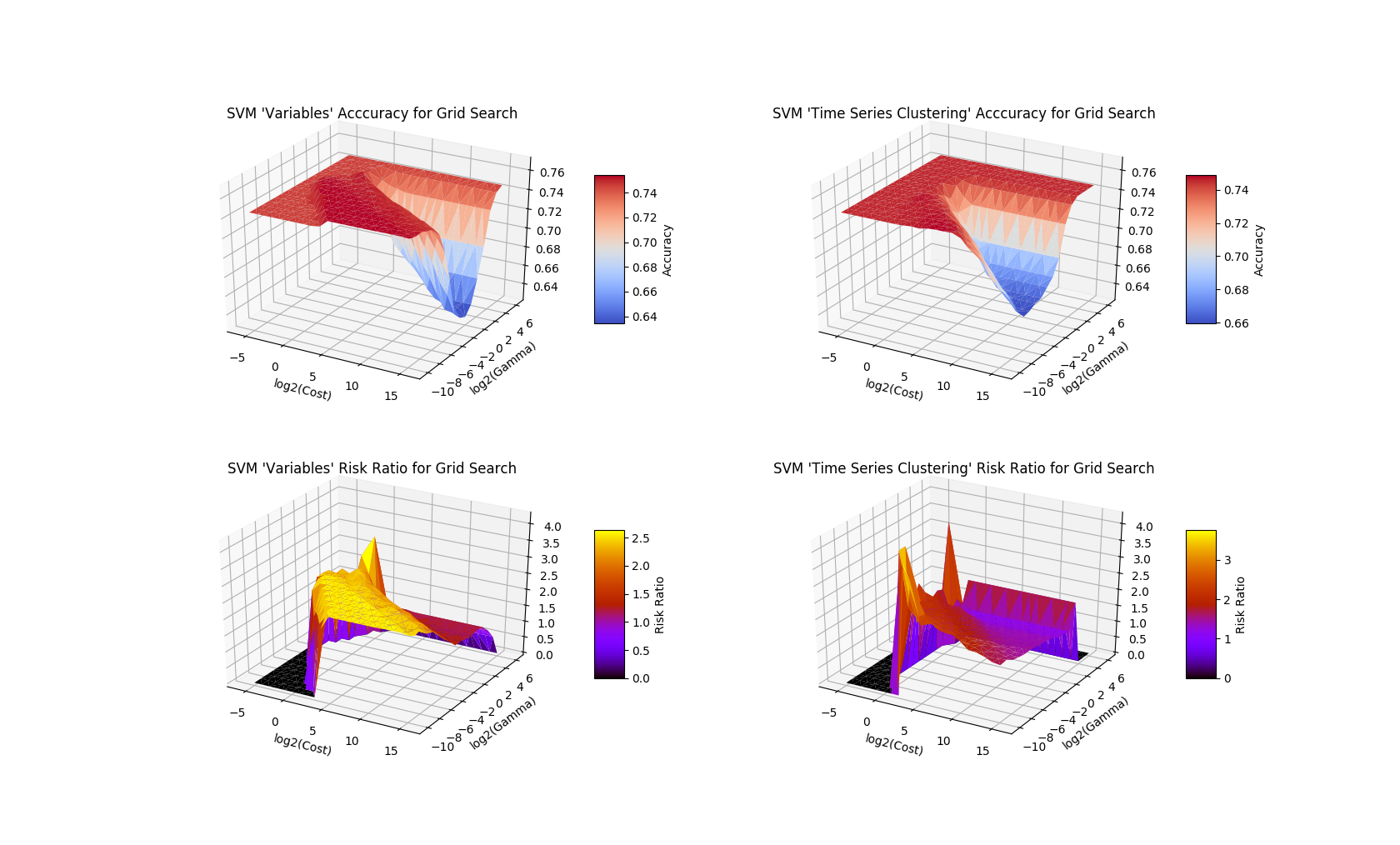
Figure 8: *We consistently observed more SVM parameterizations that better predicted ORE pass/fail using the “Variables” feature set as input than for “Time Series Clustering”. Zero is imputed for undefined Risk Ratios in these plots, such results originate from VDTs with parameterizations that fail no one.*
